# Pleuroparenchymal fibroelastosis in rheumatoid arthritis-associated interstitial lung disease

**DOI:** 10.1186/s12931-022-02064-z

**Published:** 2022-06-02

**Authors:** Jieun Kang, Woo Jung Seo, Eun Young Lee, Sung Hae Chang, Jooae Choe, Seokchan Hong, Jin Woo Song

**Affiliations:** 1 Division of Pulmonary and Critical Care Medicine, Department of Internal Medicine, Ilsan Paik Hospital, Inje University College of Medicine, Goyang, Republic of Korea; 2 Department of Pulmonary and Critical Care Medicine, Asan Medical Center, University of Ulsan College of Medicine, 88 Olympic-ro 43-gil, Songpa-gu, Seoul, 05505 South Korea; 3 Division of Rheumatology, Department of Internal Medicine, Seoul National University College of Medicine, Seoul, Republic of Korea; 4 Division of Rheumatology, Department of Internal Medicine, Soonchunhyang University Cheonan Hospital, Cheonan, Republic of Korea; 5 Department of Radiology, Asan Medical Center, University of Ulsan College of Medicine, Seoul, Republic of Korea; 6 Department of Rheumatology, Asan Medical Center, University of Ulsan College of Medicine, Seoul, Republic of Korea

**Keywords:** Pleuroparenchymal fibroelastosis, Interstitial lung disease, Rheumatoid arthritis, Mortality

## Abstract

**Background:**

Pleuroparenchymal fibroelastosis (PPFE) is a rare interstitial lung disease (ILD) featuring dense fibrosis of the visceral pleura and subpleural parenchyma, mostly in the upper lobes. PPFE can present in other ILDs, including rheumatoid arthritis-associated ILD (RA-ILD). The aim of this retrospective study was to investigate the prevalence and clinical implications of coexistent PPFE in RA-ILD.

**Methods:**

Overall, 477 patients with RA-ILD were recruited from two cohorts; their clinical data and HRCT images were analysed. The criteria for diagnosing PPFE were (1) pleural thickening with bilateral subpleural dense fibrosis in the upper lobes, (2) evidence of disease progression, and (3) absence of other identifiable aetiologies.

**Results:**

The median follow-up duration was 3.3 years. The mean age of the patients was 63.4 years, and 60.0% were women. PPFE was identified in 31 patients (6.5%). The PPFE group showed significantly lower body mass index and forced vital capacity (FVC) and more frequent usual interstitial pneumonia (UIP)-like pattern on HRCT than no-PPFE group. The risk factors for all-cause mortality were older age, lower FVC, and the presence of UIP-like pattern on HRCT; PPFE was not significantly associated with mortality in both all patients and a subgroup with a UIP-like pattern. The presence of PPFE was associated with a significantly increased risk of pneumothorax and greater decline in diffusing capacity.

**Conclusions:**

PPFE was not rare in patients with RA-ILD and was significantly associated with an increased risk of pneumothorax and greater lung function decline, though we found no significant association with mortality.

**Supplementary Information:**

The online version contains supplementary material available at 10.1186/s12931-022-02064-z.

## Background

Idiopathic pleuroparenchymal fibroelastosis (PPFE) is a rare interstitial lung disease (ILD) featuring dense fibrosis of the visceral pleura and subpleural parenchyma, with upper lobe predilection [1]. Its clinical course is heterogeneous, with some patients showing very poor outcomes owing to rapid deterioration in forced vital capacity (FVC) [2–4]. PPFE lesions can be idiopathic, but many cases occur in association with infection [5, 6], lung, bone marrow, or haematopoietic stem cell transplantation [7–9], and autoimmune diseases [10].

In recent years, there has been growing awareness of PPFE in association with other ILDs [11]. PPFE lesions have been reported in patients with idiopathic pulmonary fibrosis (IPF) [12–14] and linked to significantly higher rates of pneumothorax or pneumomediastinum [13, 14]. In another study on 359 patients with systemic sclerosis-ILD, the overall prevalence of PPFE was 18.0%, and the presence of PPFE was a significant prognostic factor for mortality [15].

Rheumatoid arthritis (RA) is a common connective tissue disease (CTD) that can be accompanied by PPFE [10]. Nevertheless, clinical implications of coexistent PPFE in RA-ILD are largely unknown. In a previous Japanese study involving 113 patients with CTD–ILD, PPFE was a significant risk factor for respiratory-related mortality [11]. However, it only included 31 patients with RA; no investigations have specifically evaluated this matter in a large cohort of patients with RA-ILD. Therefore, we evaluated the prevalence and clinical implications of PPFE in patients with RA-ILD.

## Methods

### Study patients

The study involved two cohorts of patients with RA-ILD: the Asan Medical Center (AMC) and Korean Rheumatoid Arthritis Interstitial Lung disease (KORAIL) cohorts. The AMC cohort is a retrospective cohort including 309 patients with RA-ILD (biopsy proven in 75 patients) diagnosed during January 2002–August 2018 at Asan Medical Center, Seoul, Republic of Korea. The KORAIL cohort is a prospective observational cohort including 168 patients with RA-ILD (biopsy proven in eight patients) recruited from six tertiary hospitals in the Republic of Korea (Daegu Catholic University Hospital, Kyung Hee University Hospital, Seoul National University Hospital, Seoul National University Bundang Hospital, Severance Hospital, and Soonchunhyang University Hospital) during January 2015–June 2018.

RA was diagnosed by rheumatologists based on the 2010 American College of Rheumatology/European League Against Rheumatism criteria [16], while ILD was diagnosed based on high-resolution computed tomography (HRCT) imaging and/or pathological findings. The study protocol was approved by the Institutional Review Board of Asan Medical Center (No.: 2020-0665) and Seoul National University Hospital (No.: 1801‐044‐931).

### Data collection

Baseline demographic, laboratory, pulmonary function test, and HRCT data were collected for all patients. Survival data of patients in the AMC cohort were retrospectively obtained from medical records and/or the records of National Health Insurance of Korea, whereas those of patients in the KORAIL cohort were collected prospectively.

Spirometry was performed, and the diffusing capacity of the lung for carbon monoxide (DL_CO_), as well as lung volumes, were measured according to American Thoracic Society (ATS)/European Respiratory Society (ERS) recommendations [17–19].

Data regarding pulmonary complications and follow-up pulmonary function tests were only available in the AMC cohort and obtained from records of follow-up visits (usually at intervals of 3–6 months) or hospitalisations. Pulmonary complications were categorised as pneumothorax, pneumomediastinum, acute exacerbation, pulmonary embolism, pulmonary hypertension, and lung cancer. Acute exacerbation was defined according to the criteria suggested by Collard et al., used for patients with IPF [20]. Pulmonary hypertension was defined as a maximal tricuspid regurgitation velocity of > 3.4 m/s on echocardiography, based on the 2015 European Society of Cardiology/ERS guidelines [21]. Patients’ clinical courses were followed from the diagnosis of RA-ILD until death, follow-up loss, or December 2019, whichever came first.

### HRCT evaluation

HRCT images were independently reviewed by one radiologist (J.C.) and one pulmonologist (W.J.S.). Disagreement was resolved by consensus. Inter-rater agreement for the presence of PPFE was moderate (κ statistics: 0.747). We used the clinico-radiological criteria for diagnosing PPFE previously described [14]; these were modified from the criteria published by Reddy et al. [6]. They were (i) pleural thickening with associated bilateral subpleural dense fibrosis in the upper lobes; (ii) disease progression defined as an increase in upper lobe consolidation, with or without pleural thickening and/or a decrease in upper lobe volume on serial images; and (iii) absence of other identifiable aetiologies (history of radiation therapy in the upper lung zones or active pulmonary infection). Definite PPFE was identified if all criteria were met, while possible PPFE was defined if criteria (i) and (iii) were met. Patients with RA-ILD who had definite or possible PPFE on HRCT were assigned to the PPFE group.

To determine PPFE severity, the extent of involvement was evaluated on a 4-point scale (0–3 points): 0 = absent, 1 = affecting < 10% of the pleural surface, 2 = affecting 10%–33% of the pleural surface, 3 = affecting > 33% of the pleural surface. Each of the six zones (upper, middle, and lower lung zones in the right and left lungs) was scored, and their sum was calculated. The severity was classified as either limited (≤ 2/18) or extensive (> 2/18), according to previous studies

The presence of a UIP-like pattern was assessed on HRCT and diagnosed according to the HRCT classification of the Fleischner Society IPF diagnostic guidelines, with modification [23, 24]. The UIP-like pattern was defined as a reticular pattern with peripheral traction bronchiectasis or bronchiolectasis, with or without honeycombing and without features suggesting an alternative diagnosis. In our definition of the UIP-like pattern, mosaic attenuation, air trapping, and upper- or mid-lung predominant fibrosis were not considered features of an alternative diagnosis because radiological findings of RA-ILD include mosaic attenuation or air trapping [25]. Furthermore, basal-predominant distribution, a typical feature of IPF, may not be present in RA-ILD [26]. Given that patients who have RA-ILD with a UIP pattern showed similar outcomes irrespective of whether the distribution was IPF-like or not [24], we determined UIP-like patterns without considering basal predominance in this study.

### Statistical analysis

Data are presented as percentages for categorical variables and as means ± standard deviations or medians [interquartile range] for continuous variables. Student’s *t*-test or the Mann–Whitney *U* test was used to analyse continuous variables, whereas the chi-squared and Fisher’s exact tests were used to analyse categorical variables. Binary logistic regression analysis was used to identify clinical characteristics significantly associated with PPFE. Risk factors of all-cause mortality and predictors of pneumothorax were analysed using Cox proportional hazard models. Variables with *p*-values < 0.05 in the unadjusted analyses were included in multivariable models using the enter method. Kaplan–Meier estimates and the log-rank test were used for survival analysis. The effect of time changes on pulmonary function was compared using a linear mixed model, with a covariance pattern for repeated observations. Data were analysed using the Statistical Package for the Social Sciences software version 23.0 (IBM Corp., Armonk, N.Y., USA) and R software (version 3.5.2; R Development Core Team, Vienna, Austria).

## Results

### Study patients

The study included 477 patients with RA-ILD: 309 from the AMC cohort and 168 from the KORAIL cohort. Among all patients, the mean age was 63.4 years, and female patients were predominant (60.0%). The baseline characteristics of the study patients in each hospital are described in Table S1 in Additional file 1. Patients in the AMC cohort were younger, and there were more male, ever-smokers, and seronegative patients in that cohort. With respect to lung function, patients in the AMC cohort showed significantly lower FVC, FEV_1_, and DL_CO_ than those in the KORAIL cohort. Lung volume data were not available in the KORAIL cohort. A UIP-like pattern on HRCT was more frequently found in the AMC cohort than in the KORAIL cohort (85.1% vs. 60.1%). The median follow-up duration was 3.3 years (3.9 and 2.7 years in the AMC and KORAIL cohorts, respectively).

### Prevalence and associated features of PPFE

PPFE was identified in 31 patients (6.5%) among the total cohorts (definite = 10; possible = 21); 17 of them (54.8%) had extensive PPFE. The prevalence of PPFE was 4.5% (14/309) and 10.1% (17/168) in the AMC and KORAIL cohort, respectively. Examples of limited and extensive PPFE are shown in Fig. S1 in Additional file 1. PPFE was significantly more frequent in patients with a UIP-like pattern than in those without (8.8 vs. 3.7%; p = 0.023).

Baseline characteristics of the PPFE and no-PPFE groups are compared in Table 1. The PPFE group had significantly lower mean body mass index (BMI) and FVC than the no-PPFE group, as well as higher C-reactive protein (CRP) levels and more frequent UIP-like patterns. The multivariable logistic analysis found that lower BMI and FVC were independently associated with PPFE (Table 2).

**Table 1:** Baseline characteristics of the PPFE and no-PPFE groups of patients with RA-ILD

Variables	Total	PPFE	No PPFE	p-value
Number of patients	477	31	446	
Age	63.4 ± 9.8	65.7 ± 10.6	63.2 ± 9.7	0.165
Men	191 (40.0)	12 (38.7)	179 (40.1)	> 0.999
Ever-smoker	183 (38.4)	10 (32.3)	173 (38.8)	0.568
BMI	23.6 ± 3.1	21.4 ± 2.3	23.7 ± 3.1	< 0.001
ESR	40.0 [23.0;67.0]	43.0 [26.5;75.0]	40.0 [22.0;67.0]	0.517
CRP	1.0 [0.2;4.7]	3.0 [0.6;10.7]	0.9 [0.2;4.4]	0.017
RF	391 (82.0)	28 (93.3)	363 (83.3)	0.200
RF titre	120.0 [43.7;363.0]	120.0 [39.1;439.8]	120.0 [44.3;351.5]	0.875
Anti-CCP	377 (79.0)	29 (96.7)	348 (84.5)	0.103
Anti-CCP titre	200.0 [41.8;340.0]	200.0 [60.8;559.9]	200.0 [40.5;340.0]	0.175
Pulmonary function				
FVC (%pred.)	78.0 ± 18.3	70.2 ± 20.0	78.5 ± 18.1	0.016
FEV_1_ (%pred.)	84.2 ± 21.1	79.6 ± 23.5	84.5 ± 20.9	0.220
FEV_1_/FVC	80.3 ± 8.8	82.4 ± 8.5	80.1 ± 8.8	0.171
DL_CO_ (%pred.)	66.6 ± 20.0	62.1 ± 22.5	66.9 ± 19.8	0.204
TLC^a^ (%pred.)	76.7 ± 16.1	72.8 ± 19.9	76.9 ± 16.0	0.393
RV^a^ (%pred.)	66.1 ± 20.1	69.6 ± 25.5	65.9 ± 20.0	0.548
RV/TLC^a^	0.8 [0.7;1.0]	0.9 [0.8;1.3]	0.8 [0.7;0.9]	0.084
UIP-like pattern on HRCT	260 (54.5)	23 (74.2)	237 (53.1)	0.023
Treatment				0.556
None	318 (66.7)	19 (61.3)	299 (67.0)	
Corticosteroid ± IM^b^	159 (33.3)	12 (38.7)	147 (33.0)	

Data are presented as mean ± standard deviation, median [interquartile range], or number (%), unless otherwise indicated

PPFE, pleuroparenchymal fibroelastosis; RA, rheumatoid arthritis; ILD, interstitial lung disease; BMI, body mass index; ESR, erythrocyte sedimentation rate; CRP, C-reactive protein; RF, rheumatoid factor; anti-CCP, anti-cyclic citrullinated peptide; FVC, forced vital capacity; FEV_1_, forced expiratory volume in 1 s; DLco, diffusing capacity for carbon monoxide; TLC, total lung capacity; RV, residual volume; UIP, usual interstitial pneumonia; HRCT, high-resolution chest tomography; IM, immunosuppressant

^a^Lung volume measurements were only available from patients in the AMC cohort

^b^Immunosuppresants include azathioprine (n = 47), mycophenolate mofetil (n = 43), cyclosporin (n = 7), and cyclophosphamide (n = 3)

**Table 2:** Clinical characteristics associated with PPFE in all study patients with RA-ILD

Variables	Unadjusted analysis			Multivariable analysis		
	HR	95% CI	p-value	HR	95% CI	p-value
Age	1.029	0.988–1.071	0.165			
Men	0.942	0.446–1.989	0.876			
Ever-smokers	0.751	0.346–1.634	0.471			
BMI	0.761	0.664–0.872	< 0.001	0.771	0.668–0.889	< 0.001
ESR	1.003	0.991–1.015	0.616			
CRP	1.026	1.000–1.053	0.049	1.024	0.996–1.053	0.089
FVC	0.975	0.956–0.995	0.016	0.979	0.959–1.000	0.047
DLco	0.988	0.969–1.007	0.204			
TLC	0.984	0.949–1.021	0.392			
RV	1.009	0.981–1.038	0.547			
UIP-like pattern on HRCT	3.056	0.911–10.246	0.070	2.615	0.756–9.038	0.129
Corticosteroid ± IM	1.285	0.607–2.717	0.512			

*PPFE* pleuroparenchymal fibroelastosis, *RA* rheumatoid arthritis, *ILD* interstitial lung disease, *HR* hazard ratio, *CI* confidence interval, *BMI* body mass index, *RF* rheumatoid factor, *anti-CCP* anti-cyclic citrullinated peptide, *ESR* erythrocyte sedimentation rate, *CRP* C-reactive protein, *FVC* forced vital capacity, *DLco* diffusing capacity for carbon monoxide, *TLC* total lung capacity, *RV* residual volume, *UIP* usual interstitial pneumonia, *HRCT* high-resolution chest tomography, *IM* immunosuppressant

### Survival

**Fig. 1:**
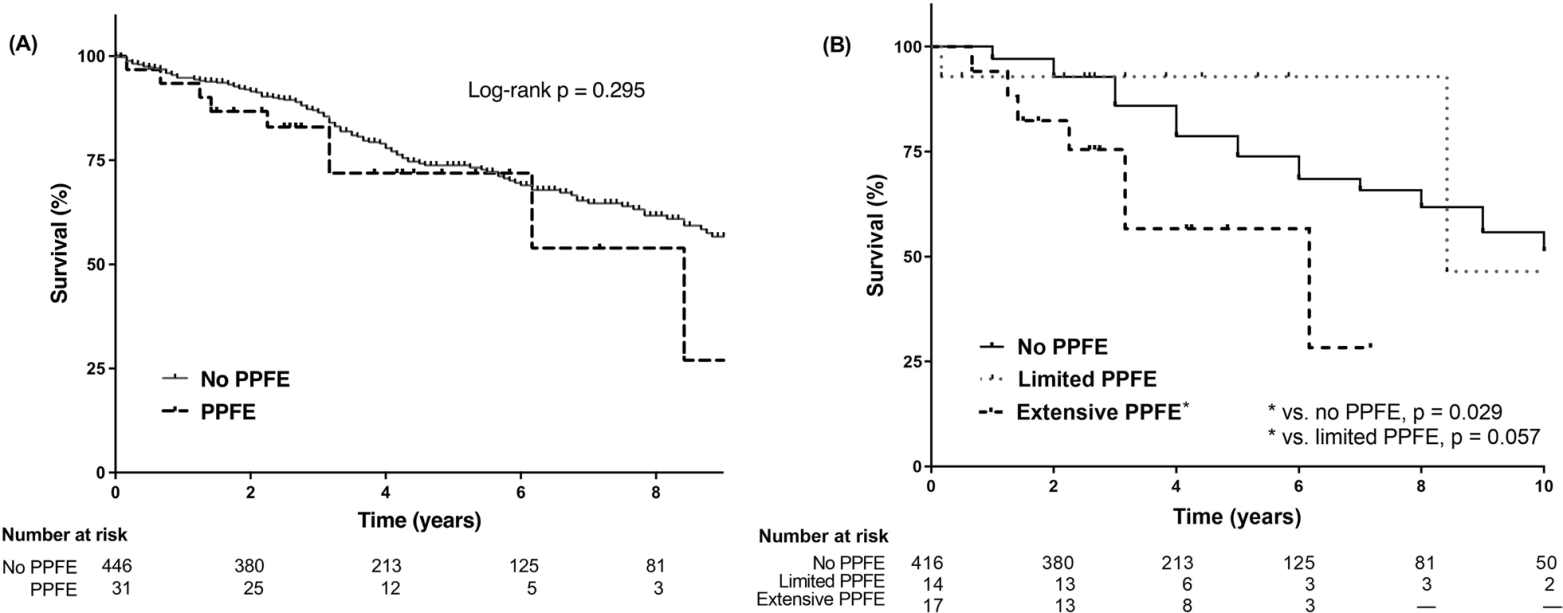
Comparison of survival between the PPFE and no-PPFE groups in patients with RA-ILD. **A** Survival curves of patients with PPFE and those without. The median survival did not significantly differ between patients with PPFE and those without (8.4 vs. 10.4 years; p = 0.295). **B** Survival curves of patients with limited and extensive PPFE, as well as those without. Patients with extensive PPFE showed a trend for worse survival (median survival: 6.2 years) than those with limited PPFE (median survival: 8.4 years; p = 0.057) and significantly worse survival than those without PPFE (median survival: 10.4 years; p = 0.029). *PPFE* pleuroparenchymal fibroelastosis

Survival analysis was performed for all cohort patients (n = 477). During the study period, 145 patients died (30.4% of all patients), and the estimated median survival was 10.3 years (95% CI: 8.4–12.3 years). There were 9 deaths in the PPFE group (29.0%) and 136 in the no-PPFE group (30.5%). Figure 1A shows the survival curves according to the presence of PPFE. Median survival did not significantly differ between patients with PPFE (8.4 years [95% CI: 3.6–13.2 years]) and those without (10.4 years [95% CI, 8.5–12.3]; p = 0.295). With regard to PPFE severity, patients with extensive PPFE showed a trend towards worse survival (median survival: 6.2 years) than those with limited PPFE (median survival: 8.4 years; p = 0.057) and significantly worse survival than those without PPFE (median survival: 10.4 years; p = 0.029), as shown in Fig. 1B.

To identify risk factors for all-cause mortality, Cox proportional hazard analyses were performed. In the unadjusted analysis, age, sex, ever-smoker, FVC, DL_CO_, a UIP-like pattern on HRCT, and extensive PPFE were significantly associated with mortality (Table 3), whereas all PPFE (limited + extensive) was not. In the multivariable model, older age, lower FVC, and a UIP-like pattern on HRCT were significant risk factors for mortality, but extensive PPFE was not.

**Table 3:** Risk factors for all-cause mortality in patients with RA-ILD

Variables	Unadjusted analysis			Multivariable analysis		
	HR	95% CI	p-value	HR	95% CI	p-value
Age	1.050	1.032–1.069	< 0.001	1.046	1.026–1.066	< 0.001
Men	1.908	1.375–2.648	< 0.001	1.919	0.964–3.819	0.063
Ever-smoker	1.543	1.113–2.138	0.009	0.799	0.400–1.596	0.525
BMI	0.953	0.903–1.005	0.074			
FVC	0.983	0.974–0.992	< 0.001	0.986	0.973–1.004	0.031
DL_CO_	0.985	0.976–0.993	0.001	0.993	0.982–1.004	0.206
TLC	0.985	0.973–0.996	0.009			
RV	0.996	0.986–1.005	0.360			
UIP-like pattern on HRCT	2.404	1.383–4.177	0.002	2.186	1.166–4.099	0.015
PPFE	1.433	0.727–2.823	0.299			
Extensive PPFE^a^	2.322	1.080–4.995	0.031	1.618	0.743–3.523	0.226
Corticosteroid ± IM	1.052	0.734–1.506	0.783			

We did not include TLC in the multivariable model, as it strongly correlated with FVC (correlation coefficient, r = 0.895; p < 0.001)

*RA* rheumatoid arthritis, *ILD* interstitial lung disease, *HR* hazard ratio, *CI* confidence interval, *BMI* body mass index, *FVC* forced vital capacity, *DLco* diffusing capacity for carbon monoxide, *TLC* total lung capacity, *RV* residual volume, *UIP* usual interstitial pneumonia, *PPFE* pleuroparenchymal fibroelastosis, *IM* immunosuppressant

^a^Extensive PPFE vs. others (limited PPFE + no PPFE)

### Clinical course and longitudinal pulmonary function changes

In the AMC cohort (n = 309), data regarding the development of pulmonary complication and sequential pulmonary function data were available. Pneumothorax occurred in four patients with PPFE (28.6%), of whom three developed recurrent pneumothorax (≥ 2 times) (Table S2 in Additional file 1). Pneumothorax was significantly more frequent in patients with PPFE than in those without (28.6% vs. 6.1%; p = 0.012). The incidence rates of pulmonary hypertension, acute exacerbation, lung cancer, or pulmonary thromboembolism were not significantly different between patients with and without PPFE. PPFE was a significant risk factor for pneumothorax (hazard ratio [HR]: 10.046, 95% confidence interval [CI]: 3.207–31.469; p < 0.001), after adjustment for sex, smoking, and DL_CO_, as shown in Table 4.

**Table 4:** Risk factors for pneumothorax in patients with RA-ILD in the AMC cohort

Variables	Unadjusted analysis			Multivariable analysis		
	HR	95% CI	p-value	HR	95% CI	p-value
Age	1.003	0.962–1.047	0.879			
Men	2.684	1.102–6.539	0.030	0.572	0.083–3.948	0.571
Ever-smokers	3.119	1.248–7.791	0.015	6.114	0.796–46.945	0.082
BMI	0.900	0.79–1.040	0.152			
FVC	0.986	0.961–1.012	0.286			
DL_CO_	0.968	0.945–0.992	0.008	0.968	0.944–0.993	0.011
TLC	0.990	0.963–1.019	0.500			
RV	0.984	0.960–1.008	0.198			
UIP-like pattern on HRCT	3.630	0.487–27.071	0.209			
PPFE	6.876	2.295–20.595	0.001	10.046	3.207–31.469	< 0.001
Corticosteroid ± IM	0.550	0.186–1.627	0.280			

*RA* rheumatoid arthritis, *ILD* interstitial lung disease, *HR* hazard ratio, *CI* confidence interval, *BMI* body mass index, *FVC* forced vital capacity, *DLco* diffusing capacity for carbon monoxide, *TLC* total lung capacity, *RV* residual volume, *UIP* usual interstitial pneumonia, *PPFE* pleuroparenchymal fibroelastosis, *IM* immunosuppressant

**Fig. 2:**
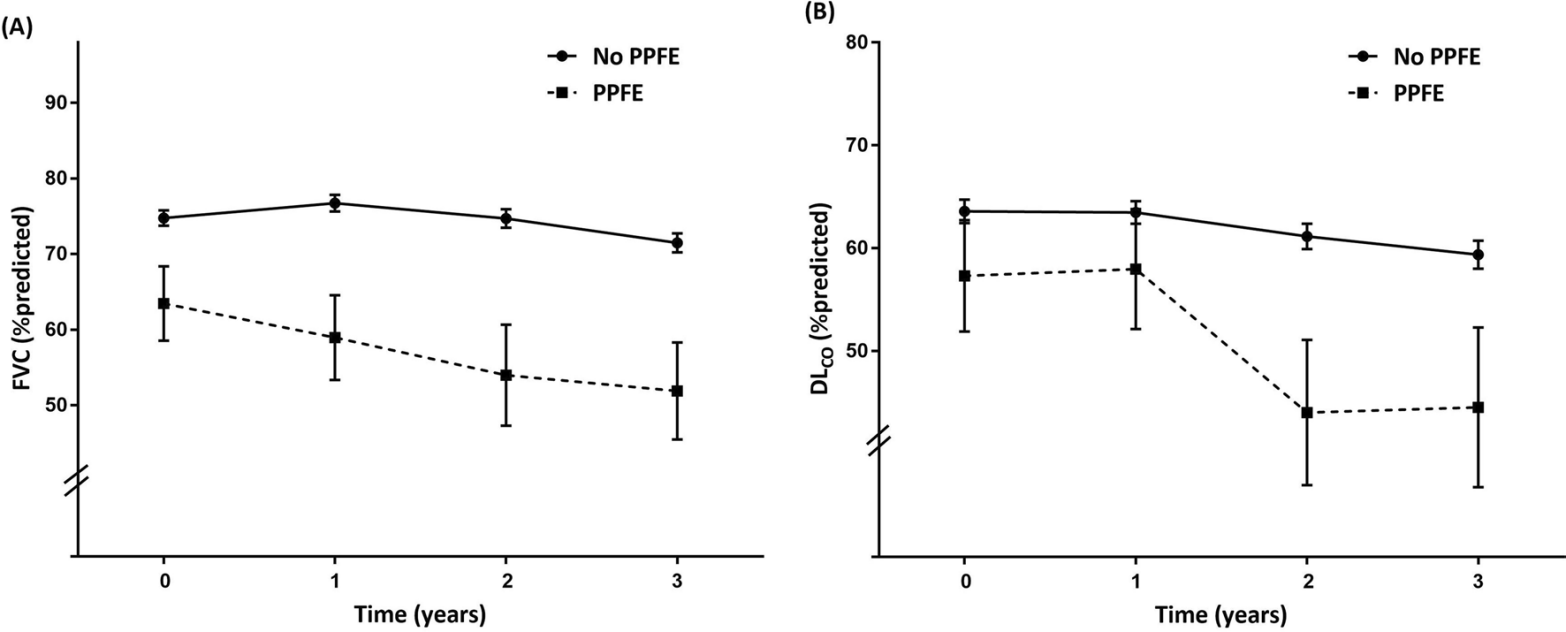
Comparison of longitudinal pulmonary function changes between the PPFE and no-PPFE groups in the AMC cohort. Changes in (A) FVC and (B) DL_CO_ are presented as least squares mean ± standard error. *PPFE* pleuroparenchymal fibroelastosis, *FVC* forced vital capacity, *DLco* diffusing capacity for carbon monoxide

Figure 2 and Additional file 1: Table S3 show longitudinal changes in FVC and DL_CO_ in the PPFE and no-PPFE groups. In the PPFE group, the decline in FVC was numerically greater and DL_CO_ was significantly greater than those in the no-PPFE group (Fig. 2A, B respectively).

### Subgroup analysis

Because a UIP-like pattern, the most common radiological pattern in RA-ILD [27], significantly influences mortality of patients with RA-ILD [28], subgroup analyses were performed according to the presence of a UIP-like pattern to determine the clinical implications of PPFE independent of the effect of a UIP-like pattern. The number of patients with a UIP-like pattern was 364, comprising 76.3% of the total cohort. Of them, 28 patients had coexistent PPFE; they showed significantly lower BMI, higher CRP level, and lower FVC than those with only a UIP-like pattern (Table S4 in Additional file 1). In this subgroup of patients, PPFE did not appear to have a significant impact on mortality. Extensive PPFE was linked to mortality in the unadjusted Cox hazard model (HR 2.142, 95% CI: 0.992–4.624; p = 0.052) but not in the multivariable analysis (Table S5 in Additional file 1). Pneumothorax developed significantly more frequently (28.6% vs. 6.8%; p = 0.018; Table S6 in Additional file 1) in patients with coexistent PPFE. After adjustment for sex, smoking, and DL_CO_, PPFE remained a significant risk factor for pneumothorax (HR 8.147, 95% CI: 2.597–25.560; p < 0.001) (Table S7 in Additional file 1). Longitudinal lung function data were available for patients with RA-UIP in the AMC cohort. Compared with the no-PPFE group, the declines of FVC and DLCO were numerically greater in the PPFE group although we did not find statistical significance (Table S5 in Additional file 1). Patients without a UIP-like pattern (n = 113) included only 3 patients with coexistent PPFE (Table S9 in Additional file 1). There was no death among these 3 patients during the follow-up period whereas 14 patients (12.7%) died in those without PPFE.

## Discussion

In this study, we analysed the prevalence and clinical implications of PPFE in patients with RA-ILD. The prevalence of PPFE was 6.5% in our study patients. Patients with PPFE had a lower BMI and FVC at baseline, higher CRP level, and more frequent UIP patterns on HRCT. Moreover, PPFE was significantly associated with increased pneumothorax risk and greater decline in lung function but not with mortality.

Idiopathic PPFE is listed as a rare idiopathic interstitial pneumonia in the classifications of the ATS/ERS [1]; however, PPFE is not rare in patients with other ILDs. In their retrospective study, Lee et al. reported that PPFE was present in 6.3% of 445 patients with IPF [14]. In another study, Oda et al. reported that 8.2% of 110 Japanese patients with IPF had biopsy-confirmed PPFE [13]. The prevalence of PPFE may vary depending on the type of ILD. In a recent study involving 359 patients with systemic sclerosis-ILD from two cohorts, the PPFE prevalence was 18.1% [15]. In another study analysing chest CT images of 233 patients with hypersensitivity pneumonitis, 23% of patients showed marked PPFE [22]. To our knowledge, the present study was the first to investigate the prevalence of PPFE in patients with RA-ILD. It remains unclear whether the prevalence of PPFE differs depending on the underlying ILD, and if so, which ILDs frequently accompany PPFE.

In this study, patients with PPFE had similar characteristics as those in previous studies, namely lower BMI and FVC [13, 14, 29, 30]. PPFE is characterised by restrictive ventilatory impairment and markedly reduced FVC that are likely caused by pleural fibrosis and thoracic cage deformity [31]. Notably, PPFE was more frequent in patients with a UIP-like pattern on HRCT in this study. Previous studies have also shown that a UIP is the commonest pattern of fibrotic ILD that coexists with PPFE, with a prevalence of 25–54% [3, 6, 29]. A UIP pattern is characterised by progressive fibrosis, mainly in the lower lobes [23], whereas PPFE mostly involves the upper lobes [1]; one may assume that coexistent PPFE and UIP pattern likely correlates with worse outcomes. In our subgroup of patients with a UIP-like pattern, extensive PPFE showed a trend towards higher mortality, though no significant impact was found in the multivariable analysis.

PPFE appeared not to influence mortality in patients with RA-ILD in the present study. The DL_CO_ decline rate was significantly greater in the PPFE group than in the no-PPFE group, though there was only a numerically greater decline in FVC. In contrast, the aforementioned study performed on patients with systemic sclerosis-ILD showed that PPFE was an independent prognostic factor; the HR for mortality was 1.89 (95% CI: 1.10–3.25), adjusted for clinical characteristics such as age, sex, treatment, and Goh staging [32]. PPFE has also been linked to a greater decline in FVC (66 mL/year vs. 44 mL/year; p = 0.08) [15]. One plausible explanation for the discrepant results is due to different types of underlying ILD. In a previous study including patients with IPF, PPFE did not significantly impact mortality, similar to the present study [14], which included patients with RA-ILD, in whom a UIP pattern was the most common radiological subtype [33–35] and a significant risk factor for mortality [28]. The impact of PPFE might have been less significant than that of the UIP pattern. We performed the subgroup analyses in patients with and without a UIP-like pattern, respectively, in order to assess the impact of PPFE independent of the influence of a UIP-like pattern. In patients showing a UIP-like pattern, the results were similar to those in all cohort patients. Patients without a UIP-like pattern included only 3 patients with PPFE and it was not possible to derive any significant results due to a very small number of patients. Further studies are warranted to investigate whether the type of underlying ILD is important in interpreting the clinical significance of PPFE.

Pneumothorax was significantly more common in patients with PPFE. Interestingly, three quarters of patients who developed pneumothorax in the PPFE group experienced recurrences (≥ 2), suggesting a strong association between PPFE and pneumothorax risk. The frequent pneumothorax in idiopathic PPFE may be caused by low resistance of the pleura to shear stress or by cysts in the apical fibrotic area [36]. Patients with PPFE showed a UIP-like pattern on HRCT more frequently than those without PPFE, and they had worse lung function; both factors may have contributed to the higher risk of pneumothorax, though PPFE was also an independent risk factor for pneumothorax in our study.

There are some limitations to be addressed. First, the present study included patients from two different cohorts. The baseline characteristics of the AMC and KORAIL cohorts were different in several parameters; patients in the KORAIL cohort were older but showed better FVC and DL_CO_, indicating less severe disease. However, various clinical features were adjusted in our Cox proportional hazard model when analysing the impact of PPFE. Furthermore, by combining heterogeneous cohorts, we believe that our study considered a broad spectrum of disease severity. Second, the KORAIL cohort provided no information on sequential pulmonary function tests or pulmonary complications. The development of pulmonary complications, such as pneumothorax or pulmonary hypertension, was only assessed using the AMC cohort data. Nevertheless, we found that the risk of pneumothorax was higher in patients with PPFE, consistent with previous reports [14, 15]. Data on the incidence of PPFE and mortality were available from both cohorts. Third, the diagnosis of PPFE was based on clinico-radiological assessments rather than histopathological findings. The radiological characteristics of PPFE are highly distinct from those of other ILDs. We attempted to exclude other possibilities, including apical cap, by setting disease progression as one of the diagnostic criteria. Furthermore, one previous study showed that the features of clinically diagnosed PPFE patients were similar to those of biopsy-confirmed PPFE patients, such as low BMI, high residual volume/total lung capacity ratio, and higher pneumothorax risk [29]. In addition, tissue biopsy is not feasible in many cases and PPFE may be identified only by HRCT. Thus, the clinico-radiological criteria can be useful in real clinical practice. Last, the number of patients with PPFE was relatively small, though we recruited a large number of patients with RA-ILD. PPFE prevalence in RA-ILD appears lower than that in systemic sclerosis-ILD [11, 15]. Nonetheless, our study has value in that it was the first to investigate the clinical impact of PPFE in RA-ILD.

In conclusion, PPFE was not rare in patients with RA-ILD, and it was significantly associated with an increased risk of pneumothorax and greater lung function decline, though there was no significant association with mortality. Further studies are needed to investigate the clinical significance of PPFE in patients with different types of ILD.

## Supplementary Information

**Additional file 1. **Additional tables and figure.

## Data Availability

Data will be available upon reasonable request. All requests should be submitted to the corresponding author for consideration.

## Abbreviations

AMC: Asan Medical Center
ATS: American Thoracic Society
BMI: Body mass index
CI: Confidence interval
CRP: C-reactive protein
CTD: Connective tissue disease
DL_CO_: Diffusing capacity of the lung for carbon monoxide
ERS: European Respiratory Society
FVC: Forced vital capacity
HR: Hazard ratio
HRCT: High-resolution computed tomography
ILD: Interstitial lung disease
IPF: Idiopathic pulmonary fibrosis
KORAIL: Korean Rheumatoid Arthritis Interstitial Lung disease
PPFE: Pleuroparenchymal fibroelastosis
RA: Rheumatoid arthritis
UIP: Usual interstitial pneumonia
