# Supplementary material for: Pleuroparenchymal fibroelastosis in rheumatoid arthritis-associated interstitial lung disease
Source: Respir Res. 2022 Jun 2;23:143. doi: 10.1186/s12931-022-02064-z (PMC9161503; doi:10.1186/s12931-022-02064-z)
Supplement: Supplementary file 1 — Additional file 1. Additional tables and figure. [file 12931_2022_2064_MOESM1_ESM.docx]

**Additional file 1**

**Pleuroparenchymal fibroelastosis in rheumatoid arthritis-associated interstitial lung disease**

Jieun Kang^1*^, Woo Jung Seo^2*^, Eun Young Lee^3^, Sung Hae Chang^4^, Jooae Choe^5^, Seokchan Hong^6^, Jin Woo Song^2^

^1^Division of Pulmonary and Critical Care Medicine, Department of Internal Medicine, Ilsan Paik Hospital, Inje University College of Medicine, Goyang, Republic of Korea

^2^Department of Pulmonary and Critical Care Medicine, Asan Medical Center, University of Ulsan College of Medicine, Seoul, Republic of Korea

^3^Division of Rheumatology, Department of Internal Medicine, Seoul National University College of Medicine, Seoul, Republic of Korea

^4^Division of Rheumatology, Department of Internal Medicine, Soonchunhyang University Cheonan Hospital, Cheonan, Republic of Korea

^5^Department of Radiology, Asan Medical Center, University of Ulsan College of Medicine, Seoul, Republic of Korea

^6^Department of Rheumatology, Asan Medical Center, University of Ulsan College of Medicine, Seoul, Republic of Korea

**Additional file 1: Table S1. Baseline clinical characteristics of all patients with RA-ILD and by hospital**

|  | AMC cohort | KORAIL cohort | p-value |
| --- | --- | --- | --- |
| Number of patients | 309 | 168 |  |
| Age, years | 61.7 ± 10.3 | 66.4 ± 8.0 | < 0.001 |
| Men | 137 (44.3) | 54 (32.1) | 0.011 |
| Ever-smoker | 139 (45.0) | 44 (26.2) | < 0.001 |
| BMI, kg/m^2^ | 23.5 ± 3.1 | 23.7 ± 3.2 | 0.439 |
| Laboratory data |  |  |  |
| ESR, mm/hr | 44.0 [25.0;75.0] | 33.5 [21.3;53.8] | < 0.001 |
| CRP, mg/dL | 0.5 [0.2;1.9] | 4.6 [1.0;10.5] | < 0.001 |
| RF positivity | 244 (79.0) | 147 (87.5) | 0.013 |
| Anti-CCP positivity | 224 (72.7) | 153 (91.6) | < 0.001 |
| RF titre, IU/mL | 151.0 [55.0;496.0] | 83.0 [25.5;211.0] | < 0.001 |
| Anti-CCP titre, U/mL | 200.0 [72.5;340.0] | 174.9 [20.2;439.5] | 0.874 |
| Pulmonary function test |  |  |  |
| FVC, %pred. | 74.2 ± 18.0 | 85.0 ± 16.8 | < 0.001 |
| FEV_1_, %pred. | 80.2 ± 19.7 | 91.5 ± 21.7 | < 0.001 |
| FEV_1_/FVC | 81.6 ± 8.4 | 77.9 ± 19.7 | < 0.001 |
| DL_CO_, %pred. | 63.4 ± 19.5 | 72.4 ± 19.7 | < 0.001 |
| TLC, %pred. | 76.7 ± 16.0 | - | NA |
| RV, %pred. | 65.4 ± 20.1 | - | NA |
| RV/TLC | 0.86 ± 0.19 | - | NA |
| UIP-like pattern on HRCT | 263 (85.1) | 101 (60.1) | < 0.001 |
| Treatment |  |  | < 0.001 |
| None | 224 (72.5) | 94 (56.0) |  |
| Corticosteroid ± IM^*^ | 85 (27.5) | 74 (44.0) |  |

Data are presented as mean ± standard deviation, median [interquartile range], or number (%), unless otherwise indicated.

RA, rheumatoid arthritis; ILD, interstitial lung disease; BMI, body mass index; ESR, erythrocyte sedimentation rate; CRP, C-reactive protein; RF, rheumatoid factor; anti-CCP, anti-cyclic citrullinated peptide; FVC, forced vital capacity; FEV_1_, forced expiratory volume in 1 second; DLco, diffusing capacity for carbon monoxide; TLC, total lung capacity; RV, residual volume; UIP, usual interstitial pneumonia; HRCT, high-resolution chest tomography; IM, immunosuppressant

^*^Immunosuppresants include azathioprine (n = 47), mycophenolate mofetil (n = 43), cyclosporin (n = 7), and cyclophosphamide (n = 3).

**Additional file 1: Table S2. Pulmonary complications in the PPFE and no-PPFE groups in the AMC cohort**

| Variables | Total | PPFE | No PPFE | p-value |
| --- | --- | --- | --- | --- |
| Number of patients | 309 | 14 | 295 |  |
| Pneumothorax | 22 (7.1) | 4 (28.6) | 18 (6.1) | 0.012 |
| Multiple pneumothorax (≥ 2 times) | 6 (1.9) | 3 (21.4) | 3 (1.0) | 0.001 |
| Pneumomediastinum | 7 (2.3) | 0 (0.0) | 7 (2.4) | > 0.999 |
| Pneumothorax or pneumomediastinum | 26 (8.4) | 4 (28.6) | 22 (7.5) | 0.029 |
| Pulmonary hypertension | 25 (8.1) | 2 (14.3) | 23 (7.8) | 0.315 |
| Acute exacerbation | 18 (5.8) | 1 (7.1) | 17 (5.8) | 0.578 |
| Lung cancer | 15 (4.85) | 0 (0.0) | 15 (5.1) | > 0.999 |
| Pulmonary thromboembolism | 1 (0.3) | 0 (0.0) | 1 (0.3) | > 0.999 |

Data are presented as number (%). The median follow-up duration of patients with PPFE and without PPFE was 30.5 months (interquartile range, 15.3–49.8 months) and 48.0 months (interquartile range, 27.0–94.0 months), respectively (p = 0.068).

PPFE, pleuroparenchymal fibroelastosis

**Additional file 1: Table S3. Comparison of lung function changes between the PPFE and no PPFE groups in the AMC cohort**

| Year | FVC (% of the predicted) | | | DL_CO_ (% of the predicted) | | |
| --- | --- | --- | --- | --- | --- | --- |
|  | PPFE | No PPFE | p-value | PPFE | No PPFE | p-value |
| 0 | 63.5 | 74.7 | 0.422 | 57.3 | 63.6 | 0.038 |
| 1 | 59.0 | 76.7 |  | 58.0 | 63.5 |  |
| 2 | 54.0 | 74.7 |  | 44.0 | 61.2 |  |
| 3 | 51.9 | 71.5 |  | 44.5 | 59.4 |  |

PPFE, pleuroparenchymal fibroelastosis; FVC, forced vital capacity; DLco, diffusing capacity for carbon monoxide

**Additional file 1: Table S4. Comparison of the baseline characteristics between the PPFE group and no-PPFE groups of patients with RA-UIP**

| Variables | Total | PPFE | No PPFE | p-value |
| --- | --- | --- | --- | --- |
| Number of patients | 364 | 28 | 336 |  |
| Age | 64.2 ± 9.6 | 65.9 ± 10.8 | 64.1 ± 9.4 | 0.330 |
| Men | 160 (44.0) | 12 (42.9) | 148 (55.0) | > 0.999 |
| Ever-smoker | 152 (41.8) | 10 (35.7) | 142 (42.3) | 0.555 |
| BMI | 23.4 ± 3.0 | 21.5 ± 2.3 | 23.6 ± 3.0 | < 0.001 |
| ESR | 40.0 [22.0;68.8] | 44.5 [25.5;78.0] | 40.0 [22.0;67.3] | 0.506 |
| CRP | 0.9 [0.2;3.5] | 3.3 [0.5;10.9] | 0.8 [0.2;3.2] | 0.006 |
| Pulmonary function test |  |  |  |  |
| FVC | 77.9 ± 18.6 | 68.1 ± 15.6 | 78.7 ± 18.4 | 0.004 |
| FEV_1_ | 84.8 ± 21.6 | 78.9 ± 21.6 | 85.3 ± 21.6 | 0.138 |
| DL_CO_ | 66.7 ± 20.4 | 60.6 ± 23.2 | 67.2 ± 20.1 | 0.105 |
| TLC | 77.6 ± 16.3 | 72.8 ± 19.9 | 77.9 ± 16.1 | 0.300 |
| RV | 66.6 ± 20.5 | 69.6 ± 25.5 | 66.5 ± 20.3 | 0.617 |
| RV/TLC | 0.8 [0.7;0.9] | 0.9 [0.8;1.3] | 0.8 [0.7;0.9] | 0.074 |
| Treatment |  |  |  | 0.829 |
| None | 257 (70.6) | 19 (67.9) | 238 (70.8) |  |
| Corticosteroid ± IM | 107 (29.4) | 9 (32.1) | 98 (29.2) |  |

Data are presented as mean ± standard deviation, median [interquartile range], or number (%), unless otherwise indicated.

PPFE, pleuroparenchymal fibroelastosis; RA, rheumatoid arthritis; UIP, usual interstitial pneumonia; BMI, body mass index; ESR, erythrocyte sedimentation rate; CRP, C-reactive protein; FVC, forced vital capacity; FEV_1_, forced expiratory volume in 1 second; DLco, diffusing capacity for carbon monoxide; TLC, total lung capacity; RV, residual volume; IM, immunosuppressant

**Additional file 1: Table S5. Risk factors of all-cause mortality in patients with RA-UIP**

|  | Unadjusted analysis | | | Multivariable analysis | | |
| --- | --- | --- | --- | --- | --- | --- |
| Variables | HR | 95% CI | p-value | HR | 95% CI | p-value |
| Age | 1.041 | 1.023–1.061 | < 0.001 | 1.037 | 1.017–1.057 | < 0.001 |
| Men | 1.837 | 1.300–2.597 | 0.001 | 2.234 | 1.084–4.603 | 0.029 |
| Ever smoker | 1.520 | 1.078–2.143 | 0.017 | 0.681 | 0.330–1.406 | 0.299 |
| BMI | 0.946 | 0.893–1.002 | 0.059 | 0.930 | 0.873–0.991 | 0.024 |
| FVC | 0.985 | 0.975–0.994 | 0.001 | 0.989 | 0.976–1.002 | 0.110 |
| DL_CO_ | 0.984 | 0.976–0.993 | < 0.001 | 0.992 | 0.981–1.003 | 0.172 |
| TLC | 0.986 | 0.974–0.997 | 0.016 |  |  |  |
| RV | 0.997 | 0.988–1.007 | 0.583 |  |  |  |
| PPFE | 1.378 | 0.697–2.725 | 0.356 |  |  |  |
| Extensive PPFE | 2.142 | 0.992–4.624 | 0.052 | 1.498 | 0.680–3.301 | 0.316 |
| Corticosteroid ± IM | 1.287 | 0.879–1.884 | 0.194 |  |  |  |

We did not include TLC in the multivariable model, as it strongly correlated with FVC (correlation coefficient, r = 0.895; p < 0.001).

UIP, usual interstitial pneumonia; RA, rheumatoid arthritis; ILD, interstitial lung disease; HR, hazard ratio; CI, confidence interval; BMI, body mass index; FVC, forced vital capacity; DLco, diffusing capacity for carbon monoxide; TLC, total lung capacity; RV, residual volume; PPFE, pleuroparenchymal fibroelastosis; IM, immunosuppressant

Variables with p < 0.1 in the unadjusted analyses were included in multivariable models to determine the impact of extensive PPFE.

**Additional file 1: Table S6. Comparison of clinical outcomes between the PPFE and no-PPFE groups of patients with RA-UIP**

| Variables | Total | PPFE | No PPFE | p-value |
| --- | --- | --- | --- | --- |
| Number of patients^a^ | 364 | 28 | 336 |  |
| All-cause mortality | 131 (64.0) | 9 (32.1) | 122 (36.3) | 0.838 |
| Number of patients (AMC cohort)^b^ | 263 | 14 | 295 |  |
| Pneumothorax | 22 (7.1) | 4 (28.6) | 17 (6.8) | 0.018 |
| Pneumomediastinum | 7 (2.3) | 0 (0.0) | 5 (2.0) | > 0.999 |
| Pulmonary hypertension | 25 (8.1) | 2 (14.3) | 17 (6.8) | 0.268 |
| Acute exacerbation | 18 (5.8) | 1 (7.1) | 14 (5.6) | 0.570 |
| Lung cancer | 15 (4.85) | 0 (0.0) | 13 (5.2) | > 0.999 |
| Pulmonary thromboembolism | 1 (0.3) | 0 (0.0) | 0 (0.0) | NA |

Data are presented as number (%).

PPFE, pleuroparenchymal fibroelastosis; RA, rheumatoid arthritis; UIP, usual interstitial pneumonia

^a^All-cause mortality was assessed for all patients with RA-UIP. The median follow-up duration of the patients with PPFE and without PPFE was 29.5 months (interquartile range, 18.0–41.3 months) and 42.5 months (interquartile range, 25.0–73.8 months), respectively (p = 0.013).

^b^Pulmonary complications were assessed in the AMC cohort because of the data availability. The median follow-up duration of the patients with PPFE and without PPFE was 30.5 months (interquartile range, 15.3–49.8 months) and 47.0 months (interquartile range, 25.5–95.5 months), respectively (p = 0.083).

**Additional file 1: Table S7. Risk factors for pneumothorax in patients with RA-UIP in the AMC cohort**

|  | Unadjusted analysis | | | Multivariable analysis | | |
| --- | --- | --- | --- | --- | --- | --- |
| Variables | HR | 95% CI | p-value | HR | 95% CI | p-value |
| Age | 0.993 | 0.950–1.039 | 0.773 |  |  |  |
| Men | 3.018 | 1.189–7.661 | 0.001 | 0.534 | 0.074–3.857 | 0.534 |
| Ever-smokers | 3.521 | 1.342–9.237 | 0.011 | 5.891 | 0.734–47.309 | 0.095 |
| BMI | 0.897 | 0.773–1.041 | 0.152 |  |  |  |
| FVC | 0.982 | 0.957–1.007 | 0.146 |  |  |  |
| DL_CO_ | 0.964 | 0.941–0.986 | 0.002 | 0.965 | 0.942–0.989 | 0.004 |
| TLC | 0.985 | 0.958–1.014 | 0.308 |  |  |  |
| RV | 0.983 | 0.960–1.007 | 0.167 |  |  |  |
| PPFE | 6.264 | 2.077–18.888 | 0.001 | 8.147 | 2.597–25.560 | <0.001 |
| Corticosteroid ± IM | 0.659 | 0.222–1.961 | 0.454 |  |  |  |

RA, rheumatoid arthritis; UIP, usual interstitial pneumonia; HR, hazard ratio; CI, confidence interval; BMI, body mass index; FVC, forced vital capacity; DLco, diffusing capacity for carbon monoxide; TLC, total lung capacity; RV, residual volume; PPFE, pleuroparenchymal fibroelastosis; IM, immunosuppressant

**Additional file 1: Table S8. Comparison of lung function changes between the PPFE and no PPFE groups in patients with RA-UIP**

| Year | FVC (% of the predicted) | | | DL_CO_ (% of the predicted) | | |
| --- | --- | --- | --- | --- | --- | --- |
|  | PPFE | No PPFE | p-value | PPFE | No PPFE | p-value |
| 0 | 63.5 | 75.7 | 0.690 | 57.3 | 67.9 | 0.340 |
| 1 | 59.0 | 78.2 |  | 58.0 | 63.4 |  |
| 2 | 54.0 | 76.5 |  | 44.0 | 61.2 |  |
| 3 | 51.9 | 73.7 |  | 44.5 | 58.6 |  |

PPFE, pleuroparenchymal fibroelastosis; RA, rheumatoid arthritis; UIP, usual interstitial pneumonia; FVC, forced vital capacity; DLco, diffusing capacity for carbon monoxide

**Additional file 1: Table S9. Comparison of the baseline characteristics between the PPFE group and no-PPFE groups of patients without RA-UIP**

| Variables | Total | PPFE | No PPFE | p-value |
| --- | --- | --- | --- | --- |
| Number of patients | 113 | 3 | 110 |  |
| Age | 60.7 ± 10.0 | 63.8 ± 10.5 | 60.4 ± 10.0 | 0.571 |
| Men | 31 (27.4) | 0 (0.0) | 31 (28.2) | 0.560 |
| Ever-smoker | 31 (27.4) | 0 (0.0) | 31 (28.2) | 0.560 |
| BMI | 23.9 ± 3.2 | 20.8 ± 3.4 | 24.0 ± 3.2 | 0.081 |
| ESR | 38.0 [23.0;63.0] | 39.0 [28.0;NA] | 38.0 [23.0;65.3] | 0.935 |
| CRP | 1.1 [0.3;5.3] | 1.0 [0.7;NA] | 1.2 [0.3;5.6] | 0.748 |
| Pulmonary function test |  |  |  |  |
| FVC | 28.4 ± 17.4 | 89.0 ± 27.0 | 78.1 ± 17.2 | 0.285 |
| FEV_1_ | 82.0 ± 19.3 | 86.0 ± 42.9 | 81.9 ±18.7 | 0.885 |
| DL_CO_ | 65.9 ± 19.0 | 75.0 ± 6.2 | 65.7 ± 19.1 | 0.404 |
| Treatment |  |  |  | 0.094 |
| None | 61 (54.0) | 0 (0.0) | 61 (55.5) |  |
| Corticosteroid ± IM | 52 (46.0) | 3 (100.0) | 49 (44.5) |  |

Data are presented as mean ± standard deviation or number (%), unless otherwise indicated.

PPFE, pleuroparenchymal fibroelastosis; RA, rheumatoid arthritis; UIP, usual interstitial pneumonia; BMI, body mass index; ESR, erythrocyte sedimentation rate; CRP, C-reactive protein; NA, not available; FVC, forced vital capacity; FEV_1_, forced expiratory volume in 1 second; DLco, diffusing capacity for carbon monoxide; IM, immunosuppressant

**Additional file 1: Figure legends**

**Additional file 1: Figure S1. Axial computed tomography showing features of (A) limited and (B) extensive PPFE**

**
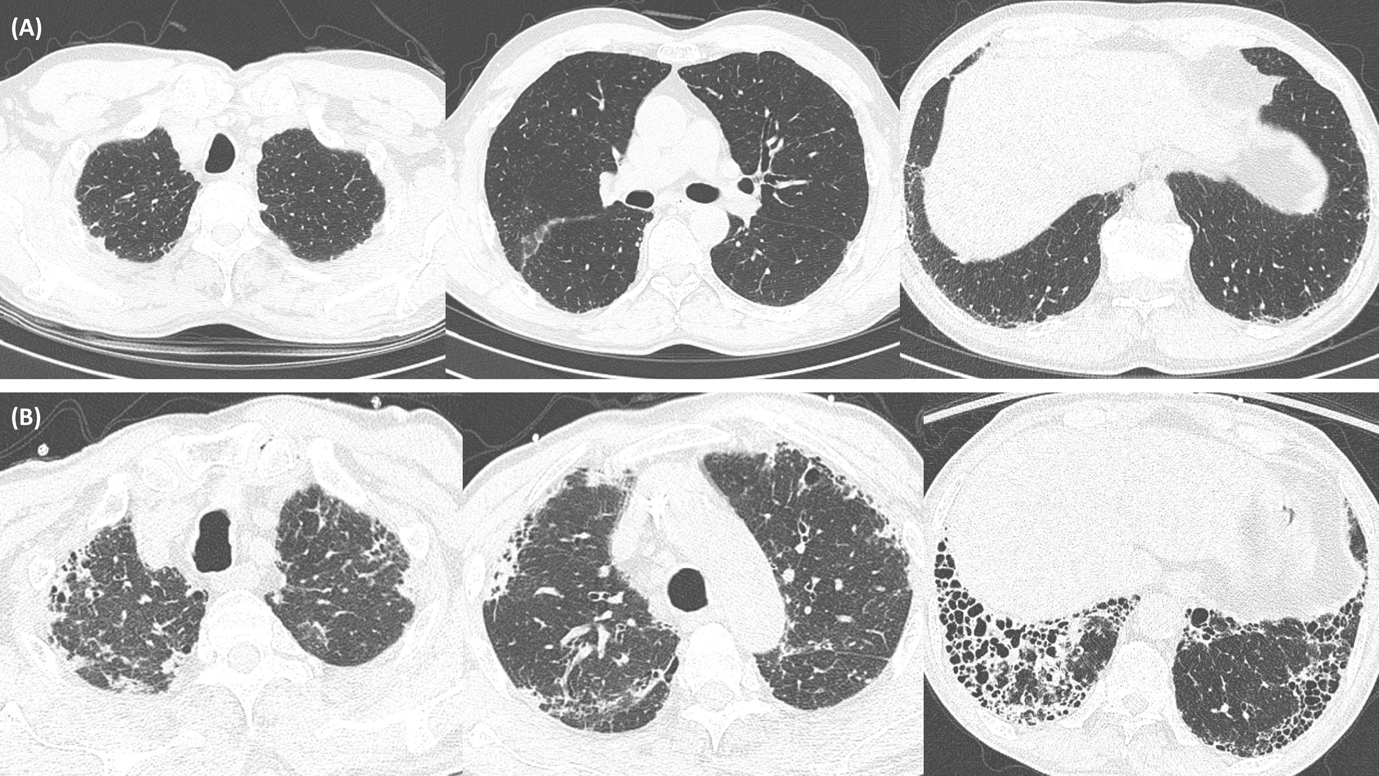
**

(A) Limited areas of pleural thickening and adjacent parenchymal fibrosis affecting less than 10% of the pleural surface in the upper lobes, and separate mild interstitial abnormalities showing reticular opacities and traction bronchiolectasis in the lower lobes. (B) Extensive areas of pleural thickening and adjacent parenchymal fibrosis affecting more than 10% of the pleural surface in the upper lobes, with traction bronchiectasis. A usual interstitial pneumonia pattern was also observed in the lower lobes.

PPFE, pleuroparenchymal fibroelastosis
